# Supplementary material for: Resonant X-ray emission spectroscopy from broadband stochastic pulses at an X-ray free electron laser
Source: Commun Chem. 2021 Jun 7;4:84. doi: 10.1038/s42004-021-00512-3 (PMC8920481; doi:10.1038/s42004-021-00512-3)
Supplement: Supplementary file 2 — Supplementary Information [file 42004_2021_512_MOESM2_ESM.pdf]

Supplementary Information

Supplementary Note 1:

Derivation of Gaussian Process for Linearly Transformed and Repeated Measurements

The RXES spectrum is a function of $\mathbf{x} = [\omega_e, \omega_i]$, where ω_i is the incident photon energy and ω_e is the emission photon energy. At a given $\mathbf{x}$, the RXES function returns a scalar intensity $y = f(\mathbf{x})$. To obtain the RXES plane, one repeatedly probes this function for a grid of input values $\mathbf{X}$ that are of interest. A Gaussian Process (GP) is a flexible distribution over scalar-valued functions that accepts multi-dimensional inputs. We can use some of its unique statistical properties to form a tractable Bayesian model to recover the RXES function from the measurements. We assume the intensity observations are corrupted by Gaussian noise $\mathbf{y} \sim N(\mathbf{f}, \sigma^2 \mathbf{I})$, a standard assumption for GP models that allows one to analytically state the conditional distribution of the RXES function given observations. In this derivation, we show how the standard GP regression setting (see Chapter 2 of Rasmussen and Williams[1]) can be adapted to linearly transformed and repeated measurements in a computationally efficient way. During the experiment, we do not observe the RXES function directly. Instead each polychromatic shot produces an intensity that is related to the RXES function as a linearly weighted sum of RXES function values: $y_i = \mathbf{w}^T f(\mathbf{X})$, where $\mathbf{w}$ is a vector representing the SASE spectral intensity for a given shot. In addition to linearly transforming the observations, there are two key constraints in this problem setting that have a large impact on computational cost. The first is that the input $\mathbf{X}$ is repeated for each shot taken since both SASE and emission spectrometers remain fixed. Second, the input $\mathbf{X}$ is as a Cartesian product over all possible incident and emission energy pairs. These aspects will be exploited later in the derivation with the consequence that training computational cost is independent of the number of shots taken.

We can show that any linear operator acting on the RXES function during measurement results only in a slight modification of the setting. A practitioner will need two expressions: the marginal likelihood, which is the objective function used to optimize hyper parameters, and the predictive distribution, which is used to plot the spectrum and error estimate. The marginal likelihood is the distribution of the observed intensities under the assumed model when all possible values of the RXES function have been integrated out. In this problem setting, the marginal likelihood is $y \sim \int df\, N(y; \mathbf{Of}, \sigma^2 \mathbf{I}) N\big(\mathbf{f}; 0, k(\mathbf{X})\big)$, where $N(\cdot; \mu, \sigma)$ is a normal distribution density, $\mathbf{O}$ is a linear operator, and $k(\cdot)$ is the kernel function (see main text). If $\mathbf{O}$ is the identity matrix, this statement of the marginal likelihood corresponds that found in the standard GP setting. With a change of variables $\mathbf{g} = \mathbf{Of}$, we can instead write $\mathbf{y} \sim \int d\mathbf{g}\, N(\mathbf{y}; \mathbf{g}, \sigma^2 \mathbf{I}) N(g; 0, \mathbf{O}k(\mathbf{X})\mathbf{O}^T)$. This integral, the convolution of two Gaussian densities, is also Gaussian, so $y \sim N(\mathbf{y}; 0, \mathbf{O}k(\mathbf{X})\mathbf{O}^T + \sigma^2 \mathbf{I})$. Under the GP model all values of the RXES function are distributed jointly as a multivariate normal for any finite set of points, which means the joint distribution of polychromatic observations $\mathbf{y}$ and RXES function values $f(\mathbf{X}_*)$ are given by:

$$\begin{bmatrix} \mathbf{y} \\ f(\mathbf{X}_*) \end{bmatrix} \sim N\left(\begin{bmatrix} 0 \\ 0 \end{bmatrix}, \begin{bmatrix} \sigma^2 \mathbf{I} + \mathbf{O}k(\mathbf{X})\mathbf{O}^T & \mathbf{O}k(\mathbf{X}, \mathbf{X}_*) \\ k(\mathbf{X}_*, \mathbf{X})\mathbf{O}^T & k(\mathbf{X}_*) \end{bmatrix} \right) \quad (1)$$

From this joint distribution, the conditional distribution of the RXES function given our linearly transformed measurements, also known as the predictive distribution, is also normally distributed. The mean and covariance of the predictive distribution is:

$$\mathbb{E}[f(\mathbf{X}_*)] = k(\mathbf{X}_*, \mathbf{X})\mathbf{O}^T[\sigma^2\mathbf{I} + \mathbf{O}k(\mathbf{X})\mathbf{O}^T]^{-1}\mathbf{y} \quad (2)$$
$$\mathbb{V}[f(\mathbf{X}_*)] = k(\mathbf{X}_*) - k(\mathbf{X}_*, \mathbf{X})\mathbf{O}^T[\sigma^2\mathbf{I} + \mathbf{O}k(\mathbf{X})\mathbf{O}^T]^{-1}\mathbf{O}k(\mathbf{X}, \mathbf{X}_*)$$

The mean and covariance functions are the best fit and error of the fit, respectively. The linear operator $\mathbf{O}$ can be broken into two linear operators, one that tiles the RXES function to reflect the repeated measurement axes for each shot, and one that applies the SASE spectral weights. To tile the RXES function evaluated on a grid of emission and incident energy positions with a linear operator n times, we left multiply by $\mathbf{T} = \mathbf{I}_q \otimes \mathbf{1}_n \otimes \mathbf{I}_r$. $\mathbf{1}_n$ is a column vector of value 1 with n rows, $\mathbf{I}_r$ is an identity matrix of size the number of pixels in the SASE spectrometer measurement, $\mathbf{I}_q$ is an identity matrix of size the number of pixels in the emission spectrometer measurement, and $\otimes$ denotes a Kronecker product. To apply SASE weights, the operator is $\widetilde{\mathbf{W}} = \mathbf{I}_q \otimes RKR\{\mathbf{I}_n, \mathbf{W}\}$, where $\mathbf{W}$ are the SASE spectral intensity measurements arranged in rows, and RKR denotes the row-partitioned Khatri-Rao[2] product. The two operators can be simultaneously applied, which results in considerable simplification:

$$\begin{aligned} \mathbf{T}\widetilde{\mathbf{W}} &= (\mathbf{I}_q \otimes \mathbf{1}_n \otimes \mathbf{I}_r)(\mathbf{I}_q \otimes RKR\{\mathbf{I}_n, \mathbf{W}\}) \\ &= \mathbf{I}_q \otimes \mathbf{W} \\ &\triangleq \mathbf{O} \end{aligned} \quad (3)$$

Equipped with this definition of the linear operator, we have a naïve set of equations which can solve the problem, but as posed both the prediction and objective function scale cubically in the number of shots, which would make it only feasible to measure around 10k shots before exhausting compute resources on a desktop machine. The cubic computational cost comes from the need to invert the matrix $\sigma^2\mathbf{I} + \mathbf{O}k(\mathbf{X})\mathbf{O}^T$ as well as to find its determinant (which appears in the evaluation of the marginal likelihood). The linear operator $\mathbf{O}$ is of size $n \times qr$ and for many more shots n than pixels in the RXES plane qr, this matrix takes on an advantageous diagonal plus low-rank matrix format. The Woodbury matrix identity[3] and determinant lemma[4] allows us to exactly re-write the inverse and determinant of a diagonal plus low-rank form matrix, respectively as:

$$\begin{aligned} \mathbf{\Sigma} &= \sigma^2\mathbf{I} + \mathbf{O}k(\mathbf{X})\mathbf{O}^T \\ \mathbf{B} &\triangleq \mathbf{I}_{rq} + \sigma^{-2}\mathbf{L}^T\mathbf{O}^T\mathbf{O}\mathbf{L} \\ \mathbf{\Sigma}^{-1} &= \sigma^{-2}\mathbf{I} - \sigma^{-4}\mathbf{O}\mathbf{L}\mathbf{B}^{-1}\mathbf{L}^T\mathbf{O}^T \\ \det(\mathbf{\Sigma}) &\triangleq \sigma^{2n}\det(\mathbf{B}) \end{aligned} \quad (4)$$

Where $\mathbf{L}$ denotes the Cholesky factor of $k(\mathbf{X})$. In this form, the computational complexity will be dominated by computing the Cholesky factorization of $\mathbf{B}$, which is $r \times q$, hence the computation is cubic in the size of the RXES plane, rather than the number of shots. With these pieces, we can state the formulae used to obtain the spectra in Figure 2 and the various simulated signal recovery scenarios. A few intermediate variables to abbreviate the equations:

$$\mathbf{L}_i \triangleq \text{cholesky}(k_i(\mathbf{X})) \quad (5)$$

$$\mathbf{C} \triangleq \mathrm{k_e}(*,\mathbf{X})\mathrm{L_e} \otimes k_\mathrm{i}(*,\mathbf{X})\mathbf{W}^T\mathbf{W}\mathbf{L}_\mathrm{i}$$

$$\begin{aligned}\mathbb{E}[f(*)] &= \sigma^{-2}k_\mathrm{e}(*,\mathbf{X}) \otimes k_\mathrm{i}(*,\mathbf{X})\mathbf{W}^\mathrm{T}\mathbf{y} \\ &\quad + \sigma^{-4}\mathrm{k}(*,\mathbf{X})\mathbf{O}^\mathrm{T}\mathbf{OLB}^{-1}\mathbf{L}^\mathrm{T}\mathbf{O}^\mathrm{T}\mathbf{y} \\ \mathbb{V}[f(*)] &= \mathrm{k_e}(*,*) \otimes \mathrm{k_i}(*,*) - \sigma^{-2}\mathrm{k_e}(*,\mathbf{X})\mathrm{k_e}(\mathbf{X},*) \\ &\quad \otimes \mathrm{k_i}(*,\mathbf{X})\mathrm{k_i}(\mathbf{X},*) + \mathbf{CB}^{-1}\mathbf{C}^\mathrm{T} \\ \mathcal{L} &= -\frac{nq}{2}\log(2\pi) - \frac{nq}{2}\log(\sigma^{-2}) - \mathrm{sumlogdiag}\big(\mathrm{cholesky}(\mathbf{B})\big) \\ &\quad - \sigma^{-2}\mathbf{y}^T\mathbf{y} + \sigma^{-4}\mathbf{y}^T\mathbf{OLB}^{-1}\mathbf{L}^T\mathbf{O}^T\mathbf{y}\end{aligned}$$

The notation $k_e(\cdot)$ indicates that a separate kernel is used for the emission energy values from the incident energy kernel $k_i(\cdot)$. This separation is valid for separable kernels, such as Squared Exponential or the Matern family of kernels. The overall kernel for a two-dimensional input is given by the product: $k_e(\cdot)k_i(\cdot)$, which for gridded inputs results in a Kronecker product: $k(\cdot) = k_e(\cdot) \otimes k_i(\cdot)$. Furthermore, we signify training energy positions always as $\mathbf{X}$ while prediction positions are noted $*$. These positions may be the same or different from one another. Note that terms that scale with the number of shots n like $\mathbf{W}$ or $\mathbf{y}$ always appear as either $\mathbf{W}^T\mathbf{y}$ or $\mathbf{W}^T\mathbf{W}$, which means that these terms can be pre-compute and cached – eliminating any scaling of the predictive distribution $f(*)$ or the objective function $\mathcal{L}$ on the number of shots.

Supplementary Figure 1: Impact of longer measurement for stochastic spectroscopy

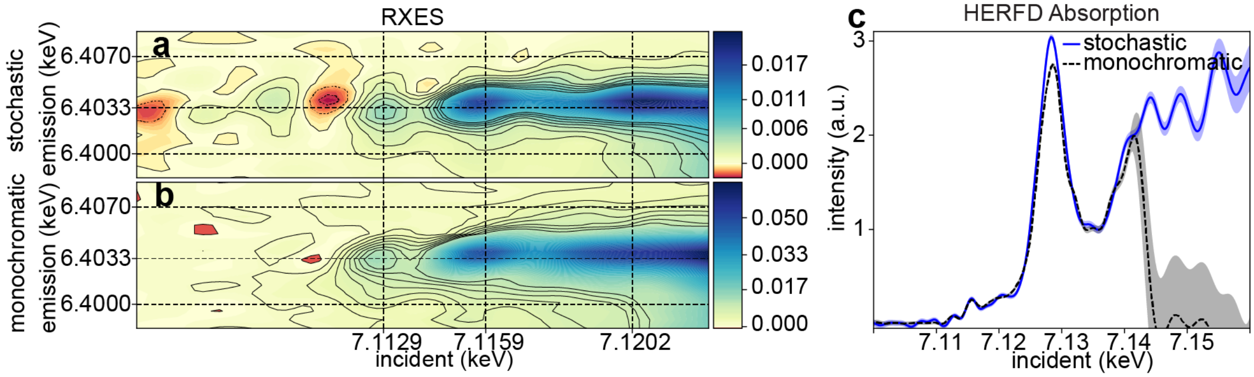

As for **Figure 1**, in **panel a**, the recovered RXES plane of the $K\alpha_1$ line in the pre-edge region from polychromatic beam measurements for 101,000 shots is compared to a monochromatic measurement with 42,000 shots in panel **b**. Solid black contour lines indicate an increase of RXES intensity by 2σ, where σ is the standard deviation of the predictive distribution estimated for a given emission/incident energy point. In **panel c**, a slice at constant emission energy (6.4033 keV) through the RXES plane is shown spanning a wider range of incident energies than in **panels a** and **b**.

Supplementary Figure 2: Corrective effects applied to monochromatic data

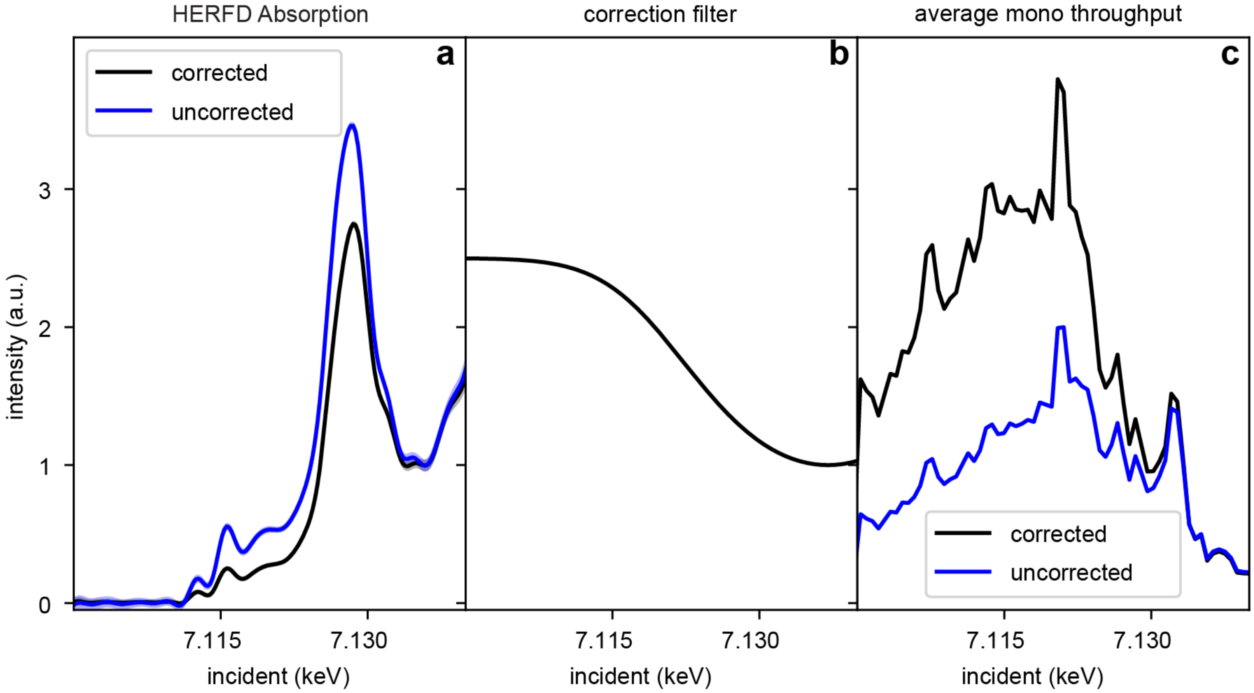

In **Figure 1**, the corrected monochromatic signal is shown. The effect of the correction is shown in panel **a**. The correction mainly affects contrast between features from 7.115-7.120 keV compared with the peak near 7.130 keV. The shape of the filter, shown in panel **b**, was optimized to match reference measurements of the same compound[5]. The shape of the filter applied, and the fact that the polychromatic measurements required no correction to match reference spectra, suggests the loss of contrast is due to a shift of beam spatial intensity distribution upon changing from SASE to monochromatic beam. Panel **c** depicts the average SASE spectrum transmitted through all monochromator settings before and after the corrective filter is applied.

Supplementary Figure 3: Simulation details

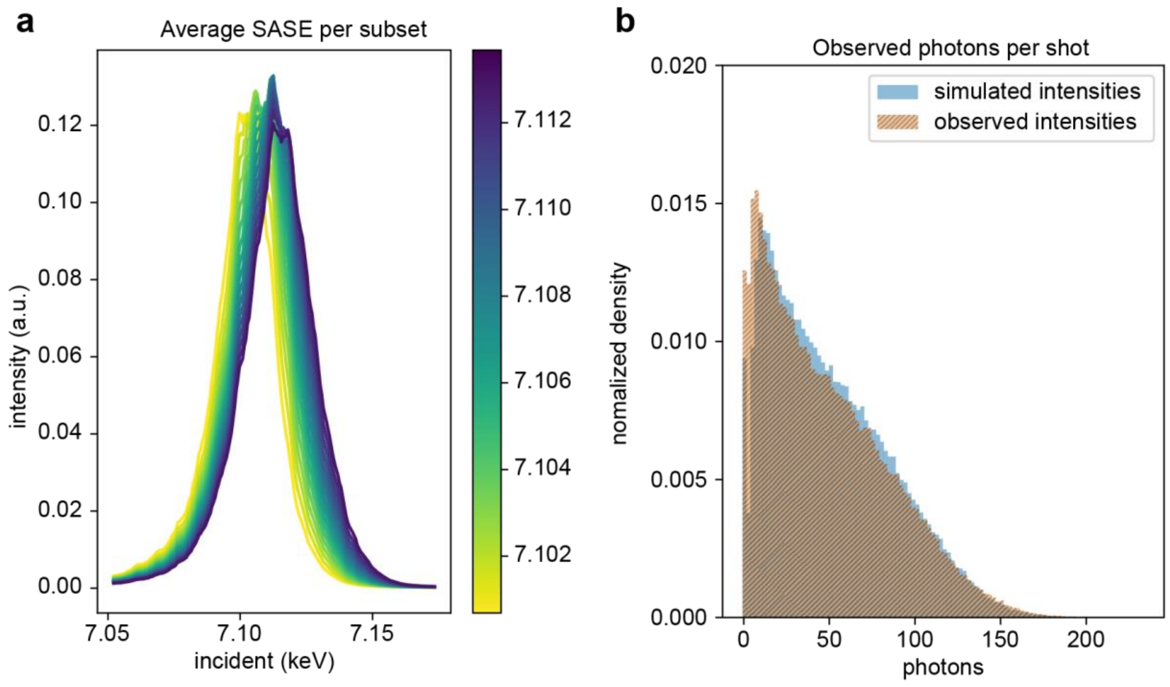

In panel **a**, the average SASE spectrum for each of the 17 independent subsets studied in **Figure 2** is shown, with their lines colored the same as in **Figure 2**. In panel **b**, the intensity distribution over all shots of the simulated data is compared to the experimentally measured through an empirical histogram.

Supplementary References

1. Rasmussen, C. E. & Williams, C. K. I. *Gaussian processes for machine learning. MIT Press* (2006). doi:10.1142/S0129065704001899.
2. Khatri, C. G. & Rao, C. R. *Solutions to Some Functional Equations and Their Applications to Characterization of Probability Distributions*. *The Indian Journal of Statistics, Series A* vol. 30 (1961).
3. Hager, W. W. Updating the Inverse of a Matrix. *SIAM Review* **31**, 221–239 (1989).
4. Kato, T. *Perturbation theory for linear operators*. (Springer Science & Business Media, 2013).
5. Penfold, T. J. *et al.* X-ray spectroscopic study of solvent effects on the ferrous and ferric hexacyanide anions. *Journal of Physical Chemistry A* **118**, 9411–9418 (2014).
